# Dunye Guanxinning Improves Acute Myocardial Ischemia-Reperfusion Injury by Inhibiting Neutrophil Infiltration and Caspase-1 Activity

**DOI:** 10.1155/2018/4608017

**Published:** 2018-02-20

**Authors:** Q. G. Zhang, S. R. Wang, X. M. Chen, H. N. Guo, S. Ling, J. W. Xu

**Affiliations:** Institute of Interdisciplinary Medical Science, Shanghai University of Traditional Chinese Medicine, Shanghai 201203, China

## Abstract

Acute myocardial infarction is the most serious manifestation of cardiovascular disease, and it is a life-threatening condition. Dunye Guanxinning (DG) is a protective traditional Chinese patent herbal medicine with high clinical efficacy and suitable for the treatment of myocardial infarction. However, the mechanism through which it is beneficial is unclear. In this study, we hypothesized that DG improves acute myocardial ischemia-reperfusion injury by inhibiting neutrophil infiltration and caspase-1 activity. We found that DG administration decreased infarct size and cardiomyocyte apoptosis and improved left ventricular ejection fraction, fractional shortening, end-systolic volume index, end-systolic diameter, and carotid arterial blood flow output in rats. DG administration also improved hemorheological parameters, myocardial damage biomarkers, and oxidative stress indexes. The findings showed that DG administration inhibited neutrophil infiltration and reduced the serum interleukin-1 beta (IL-1*β*) level and myocardial IL-1*β* maturation. Moreover, DG administration inhibited caspase-1 activity and activated adenosine monophosphate-activated protein kinase (AMPK) phosphorylation in rat hearts. These results suggested that DG administration inhibits inflammasome activity and IL-1*β* release through the AMPK pathway. Our findings support the clinical efficacy of DG and partially reveal its mechanism, which is beneficial for understanding the therapeutic effects of this protective traditional Chinese patent drug.

## 1. Introduction

Myocardial infarction is the leading cause of death worldwide, and it is associated with sudden death and is thus a serious threat to life and health. Myocardial ischemia-reperfusion (I/R) injury is the recovery of myocardial ischemia after a short period, resulting in further deterioration of myocardial function. The pathogenesis of myocardial infarction involves many factors. I/R injury first triggers neutrophil infiltration [1, 2] and subsequently initiates the generation of a large number of oxygen-free radicals [3, 4], primes the inflammatory response including inflammasome activation [5–7], induces apoptosis [8, 9], and so on. Myocardial I/R injury is also one of the important reasons for the failure of clinical thrombolytic therapy, coronary artery bypass grafting, and heart transplantation.

Dunye Guanxinning (DG) is a class B protective traditional Chinese patent drug in the Chinese Pharmacopoeia [10]. This drug is an extract obtained from the dried rhizomes of *Dioscorea zingiberensis* C. H. Wright, and it is used to treat hyperlipidemia, coronary heart disease, and angina and to improve symptoms of chest tightness, palpitation, dizziness, and insomnia [10]. The fresh rhizome contains various saponins such as Huangjiangsu A, zingiberensis new saponin, deltonin, dioscin, and gracillin (Table 1) [11]. Clinical investigations have found that DG can effectively lower the blood lipid level [12], decrease angina attack frequency, shorten angina onset time, and improve endothelial function and blood rheology [13, 14]. DG also effectively reduces serum homocysteine levels in patients with ischemic heart disease [15]. The combination of DG with other traditional patent drugs has been demonstrated to decrease the incidence of angina pectoris, reinfarction, cardiac insufficiency, and platelet activation in patients with acute myocardial infarction [16]. DG effectively improves angina and myocardial infarction clinically; however, the mechanism by which it achieves this is unclear. Therefore, in this study, we used a myocardial I/R model to verify whether DG improves myocardial I/R injury by inhibiting neutrophil infiltration and inflammasome NLR family pyrin domain containing 3 (NLRP3) activity, providing a reliable basis for interpreting the mechanism of DG.

## 2. Materials and Methods

### 2.1. Animal Ethics Statement and Animals

The animals received care in compliance with the Guide for the Care and Use of Laboratory Animals published by the US National Institutes of Health, and the animal experiments in this study were approved by the Animal Ethics Committee of Shanghai University of Traditional Chinese Medicine (approval number SZY201510001).

Male Sprague–Dawley (SD) rats aged 7-8 weeks were purchased from Shanghai SIPPR-BK Lab Animal Co. Ltd. (Shanghai, China). Rats were housed in cages (three per cage) maintained at constant humidity (65% ± 5%) and temperature (24°C ± 1°C) in a 12-hour light-dark cycle. Rats were allowed ad libitum access to tap water and food throughout the experimental protocols. Thirty-six male SD rats were randomly divided into the DG control, sham operation, I/R, and I/R + DG groups (12 per group). Rats in the I/R + DG group were treated with DG for 2 weeks (100.8 mg/kg/day, changed according to the clinical dose). The equivalent dose in rats of 100.8 mg/kg/day was calculated as 960 mg/day (human clinical dose)/60 kg (normal Chinese human body weight) × 6.3 (equivalent dose ratio of rats to humans); the equivalent dose ratio (6.3) was calculated from the conversion factor (0.018) of the human clinical dose to the effective rat dose. By contrast, rats in the sham operation group and the I/R group were given an equal volume of pure water. On the last day of treatment, rats were anesthetized using an intraperitoneal injection of sodium pentobarbital at a dose of 45 mg/kg, and their organs were dissected and isolated. After the organs were weighed, they were frozen in liquid nitrogen and stored at −80°C.

### 2.2. Myocardial Ischemia-Reperfusion Model

After rats were anesthetized using sodium pentobarbital, they were placed on an electric heating pad to maintain their body temperature at 37°C and were artificially ventilated using a volume-controlled RWD 407 rodent respirator (Shenzhen Reward Life Technology Co. Ltd., Shenzhen, China) with the respiration rate set at 70/min, tidal volume at 1.5 mL, and breathing time ratio at 1 : 1. Animal electrocardiogram (ECG) was monitored using a PowerLab 8-channel multifunctional physiological recorder (AD Instruments, Bella Vista, Australia). The left anterior descending coronary artery was ligated for 30 min, and significant elevation of the ST segment on the ECG was used as the index of successful ligation. After ST elevation detection, the blood vessel was expanded to allow reperfusion for 2 h. Sham-operated rats underwent similar procedures without ligation of the coronary arteries.

### 2.3. Left Ventricular Function Echocardiography

Twenty-four hours after reperfusion, rats were anesthetized using 1.5% isoflurane. Their left ventricular function parameters, such as left ventricular ejection fraction (LVEF), left ventricular fractional shortening (LVFS), end-systolic volume index, and end-systolic diameter (ESD), were determined using a Vevo 770® High-Resolution Imaging System (VisualSonics Inc., Toronto, Ontario, Canada).

### 2.4. Western Blot Assay

The left ventricular tissue was lysed in ice-cold radioimmunoprecipitation assay buffer (50 mmol/L Tris/HCl, pH 8.0; 150 mmol/L NaCl; 2 mmol/L sodium orthovanadate; 1% Nonidet-P40, 1% sodium deoxycholate; 0.1% sodium dodecyl sulfate (SDS); 0.1 mmol/L dithiothreitol; 0.05 mmol/L phenylmethylsulfonyl fluoride; 0.002 mg/mL aprotimin; and 0.002 mg/mL leupeptin). Lysates were precleared through centrifugation at 12000 ×g for 10 min at 4°C. Aliquots of the cell lysate (50 or 100 *μ*g of each sample) were resolved on SDS-polyacrylamide gel electrophoresis and were transferred to nitrocellulose membranes. The membranes were blocked in 5% skimmed milk overnight at 4°C. The membranes were incubated with primary antibodies for 2 h and then incubated with an HRP-conjugated secondary antibody at room temperature for 1 hour. The bands were visualized using an ECL Immobilon Western Chemiluminescent HRP substrate (Millipore, Billerica, MA, USA). Quantitative analysis of band density was performed using Quantity One software from Bio-Rad (Hercules, CA, USA). Western blot experiments were performed in triplicate [17].

### 2.5. Immunohistochemical Staining

Formalin-fixed and paraffin-embedded tissue section blocks were cut in 6 *μ*m sections, followed by immunohistochemical staining with primary antibodies of anti-cleaved caspase-3 (GB13009 Whan Servicebio Technology Co. Ltd., Wuhan, China), anti-CD68 (GB13067-1, Servicebio), anti-MPO (GB13224, Servicebio), or anti-CD15 (GTX37536, GeneTex, Irvine, USA) and goat anti-mouse or anti-rabbit IgG secondary antibodies. The EnVision (Peroxidase/DAB, Rabbit/Mouse) detection system was used to evaluate the immunohistochemical staining. TUNEL staining was performed using the In Situ Cell Death Detection Kit (11684817910, Roche, Mannheim, Germany). The intensity of the immunohistochemical staining was analyzed using the ImageJ analysis system (NIH, Maryland, USA).

#### 2.5.1. ELISA Assay

The ELISA assay was performed using the rat IL-1*β* ELISA kit (ab100768, Abcam, Cambridge, UK) or rat interleukin-1*β*, rat interleukin-6, and rat interleukin-18 ELISA assay kits (H002, H007, and H015, Nanjing Jiancheng Bioengineering Institute, Nanjing, China) according to the manufacturers' instructions.

### 2.6. Determination of Hemodynamic Parameters

After 30 min of myocardial ischemia and 2 h after reperfusion, rats were anesthetized using an intraperitoneal injection of 1% sodium pentobarbital (3 mL·kg^−1^), and the left common carotid artery was detached. Carotid hemodynamics were recorded using the PowerLab 8-channel multifunctional physiological recorder (AD Instruments).

### 2.7. Detection of Hemorheological Parameters

Two hours after reperfusion, rats were anesthetized using an intraperitoneal injection of 1% sodium pentobarbital (3 mL·kg^−1^). A total of 5 mL of blood was collected through the aorta ventralis and was rapidly transferred to a tube containing the anticoagulant heparin. Subsequently, blood rheology was detected using an MEN-C100A automatic blood rheological dynamic analyzer (Shandong Meiyilin Electronic Instrument Co. Ltd., Jinan, China).

### 2.8. Preparation of Myocardial Tissue Supernatants and Myocardial Enzyme Activity Assay

Cardiac tissue samples in a tissue weight/normal saline ratio of 1 : 9 were placed in a liquid-nitrogen-cooled JXFSTPRP-192 automatic fast sample mill (Shanghai Jingxin Industrial Development Co. Ltd., Shanghai, China) and were ground (frequency of 75 for 50 s) twice. The tissue homogenates were removed and sonicated five times every 5 s at 10 s intervals in an ice bath on a JY92-2D ultrasonic homogenizer (NingBo Scientz Biotechnology Co. Ltd., Zhejiang, China). The samples were subsequently centrifuged at 2500 rpm for 10 min at 4°C. The separated tissue supernatants were extracted, and their protein concentrations were measured using Coomassie brilliant blue. The supernatants obtained after centrifugation of the myocardial tissue were diluted 10-fold, packed, and placed in a −80°C freezer.

Using procedures specified by the manufacturer, we determined LDH activity, CK-MB activity, catalase activity, GSH-PX activity, and GSH content using the appropriate test kits. The ideal sample concentration and measurement condition were identified according to a response curve.

### 2.9. Statistical Analysis

Data were analyzed using SPSS (SPSS Inc., Northampton, USA), and the means and standard deviations of the data were calculated. Pairwise comparisons were made using the Mann–Whitney *U* test, and comparisons were made between treatment groups by using analysis of variance and post hoc tests. *P* < 0.05 was considered statistically significant.

## 3. Results

**Figure 1:**
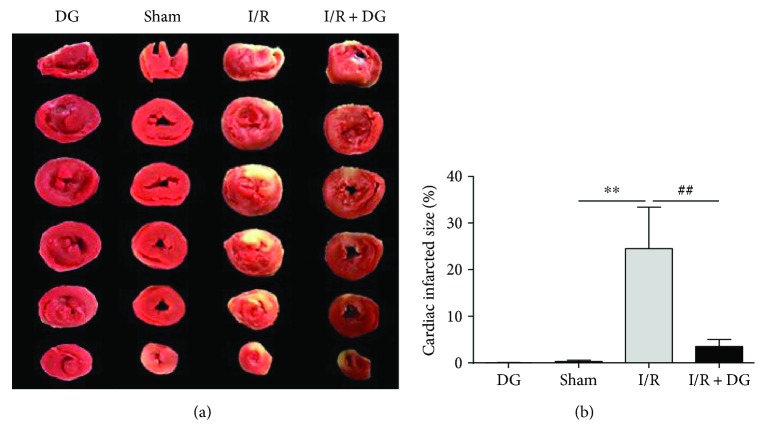
Dunye Guanxinning reduced cardiac infarct size. (a) 2,3,5-Triphenyltetrazolium chloride (TTC) staining of heart slices; (b) sizes of myocardial infarcts presented in Figure 2(a) (*n* = 6 or 7 each). Data are expressed as mean ± SD. ^∗∗^*P* < 0.01 versus sham operation group; ^##^*P* < 0.01 versus I/R group.

**Figure 2:**
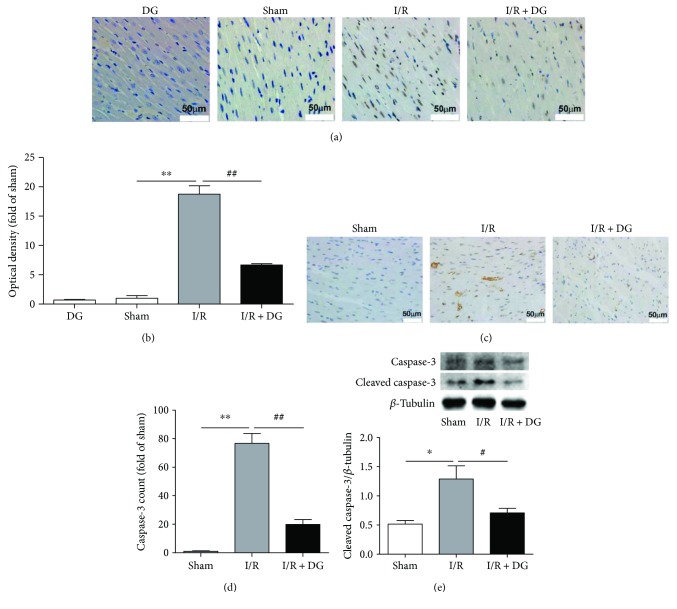
Dunye Guanxinning inhibited myocardial apoptosis. (a) Immunochemical TUNEL staining; (b) multiples of optical density of TUNEL staining from Figure 2(d) (*n* = 3); (c) immunochemical staining of anti-caspase-3 (cleaved) antibody; (d) multiples of cleaved caspase-3 staining positive cells from Figure 2(a) (each *n* = 3); (e) caspase-3 immunoblot of left ventricular tissue (each *n* = 4). Data are expressed as mean ± SD. ^∗^*P* < 0.05 or ^∗∗^*P* < 0.01 versus sham operation group; ^#^*P* < 0.05 or ^##^*P* < 0.01 versus ischemia-reperfusion group.

As shown in Figure 1, TTC staining revealed that the myocardial infarct size was significantly higher in the myocardial I/R group, reaching 24.51% ± 8.91%, than in the sham operation group (each *n* = 6 or 7, *P* < 0.01). By contrast, the prophylactic administration of DG for 2 weeks resulted in a significantly smaller myocardial infarction area of 3.52% ± 1.48% (each *n* = 6 or 7, *P* < 0.01). To determine the type of tissue death, tissue staining was performed using the cleaved caspase-3 antibody. The results showed that the number of cleaved caspase-3 stained positive cells in the myocardial infarction area of the I/R group was significantly higher (76.72% ± 11.89%) than that of the sham operation group. However, the results of section staining revealed that the apoptotic staining density of TUNEL was 18.74 ± 1.43-fold greater than that in the sham operation group (1.00 ± 0.46, each *n* = 3, *P* < 0.01; Figures 2(a) and 2(b)). The apoptotic staining optical density in the DG administration group was reduced by 6.68 ± 0.21-fold and was significantly lower than that in the I/R group (*n* = 3, *P* < 0.01; Figures 2(a) and 2(b)). Moreover, DG administration also resulted in a decrease in the number of cleaved caspase-3 stained positive cells to 20.21% ± 5.35% (*n* = 3, *P* < 0.01; Figures 2(c) and 2(d)). Western blotting was used to examine protein expression. The expression of cleaved caspase-3 in the myocardial I/R group was a factor of 2.47 ± 0.85 higher than that in the sham operation group. By contrast, the expression of cleaved caspase-3 in the DG administration group was a factor of 1.36 ± 0.28 lower than that in the I/R group (each *n* = 3, *P* < 0.05; Figure 2(e)). These results demonstrated that DG reduces myocardial damage and apoptosis caused by I/R injury.

**Figure 3:**
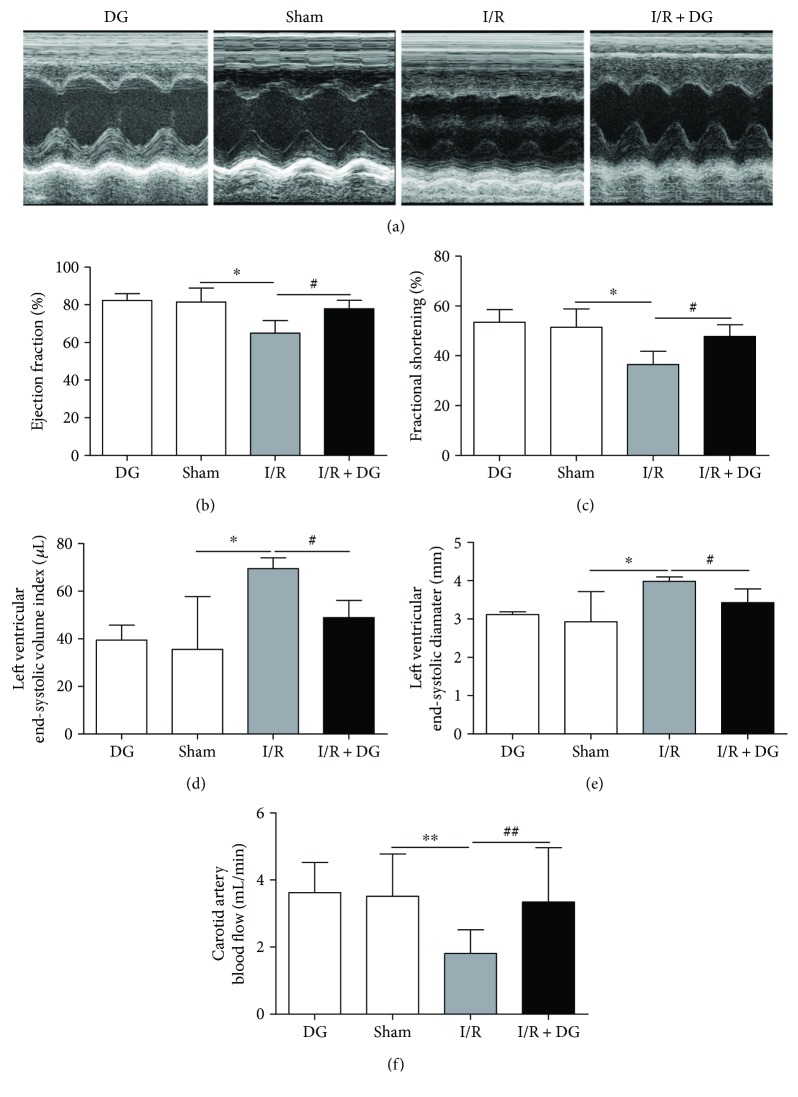
Dunye Guanxinning improved cardiac function. (a) Typical left ventricular echocardiography in rats; (b–e) LVEF, fractional shortening, end-systolic volume index, and end-systolic diameter were determined using a Vevo 770 high-resolution imaging system to measure cardiac function (*n* = 3); (f) carotid arterial blood flow was tested using a PowerLab 8-channel multifunctional recorder (*n* = 10). Data are expressed as mean ± SD. ^∗^*P* < 0.05 or ^∗∗^*P* < 0.01 versus sham operation group; ^#^*P* < 0.05 or ^##^*P* < 0.01 versus ischemia-reperfusion group.

To analyze the effect of DG administration on cardiac function, we used the Vevo Visual 770 Sonics small animal imaging system to evaluate cardiac function on 24 ± 2 h after I/R injury. The ultrasonic reflected wave of the left ventricular wall in the I/R group was flat, suggesting that the left ventricular wall was damaged and the I/R injury model was successfully established. By contrast, the reflected wave had a wave pattern in the I/R + DG group (Figure 3(a)). In the I/R group, the LVEF and fractional shortening decreased to 64.99% ± 6.66% and 36.52% ± 5.29%, respectively (*n* = 3, *P* < 0.05; Figures 3(b) and 3(c)), and the end-systolic volume index and ESD of the left ventricle increased to 69.49 ± 4.54 *μ*L and 3.98 ± 0.11 mm%, respectively (*n* = 3, *P* < 0.05; Figures 3(d) and 3(e)). By contrast, in the I/R + DG group, the LVEF and fractional shortening increased to 77.88% ± 4.49% and 47.73% ± 4.72%, respectively, and the end-systolic volume index and ESD were restored to 48.86 ± 12.69 *μ*L and 3.42 ± 0.35 mm, respectively (*n* = 3, *P* < 0.05; Figures 3(d) and 3(e)). Blood flow in internal carotid arteries was also determined. The results showed that carotid arterial blood flow decreased from 3.36 ± 1.16 mL/min in the sham operation group to 1.71 ± 0.61 mL/min in the I/R group. However, DG administration restored the blood flow to 3.03 ± 1.32 mL/min (*n* = 10, *P* < 0.05; Figure 3(f)). These results demonstrated that DG improves the recovery of cardiac function after I/R injury.

Subsequently, myocardial CK-MB and LDH activities in the rat serum were measured. The results revealed that CK-MB activity in the I/R group was significantly higher at 1.56 ± 0.72 U/mL compared with that in the sham operation group (0.83 ± 0.28 U/mL, *n* = 10, *P* < 0.01; Table 2). By contrast, the serum CK-MB activity in the I/R + DG group was significantly lower at 0.92 ± 0.29 U/mL than that in the I/R group (*n* = 10, *P* < 0.01; Table 2). Serum LDH activity exhibited a trend similar to that of CK-MB activity. Serum LDH activity in the I/R group was significantly higher at 5827.39 ± 1871.24 U/L compared with that in the sham operation group (3365.37 ± 1357.24 U/L). By contrast, serum LDH activity in the DG administration group was lower at 4112.27 ± 1165.87 U/L (each *n* = 10, *P* < 0.01; Table 2). We also measured GSH-Px activity and the GSH content in the myocardium. In the I/R group, GSH-Px activity was decreased from 320.27 ± 76.48 U/mg (protein) to 241.07 ± 76.67 U/mg (protein), and the GSH content was decreased from 12.15 ± 5.66 *μ*mol/g protein to 6.84 ± 1.53 *μ*mol/g protein (each *n* = 10, *P* < 0.01; Table 2). DG administration increased GSH-Px activity and the GSH content in the myocardium to 327.53 ± 62.91 U/mg (protein) and 14.40 ± 10.32 *μ*mol/g (protein), respectively (each *n* = 10, *P* < 0.05; Table 2). These results confirmed the effect of DG on changes in myocardial injury biomarkers and oxidative stress.

Because hemorheological parameters have been found to be associated with I/R [18–20], we also measured whole blood viscosity (WBV), erythrocyte sedimentation rate (ESR), and hematocrit (Htc). WBV at shear rates of 200 s^−1^, 50 s^−1^, and 10 s^−1^ was discovered to increase significantly from 3.52 ± 0.29 mPa·s/200 s^−1^, 3.88 ± 0.35 mPa·s/50 s^−1^, and 4.71 ± 0.48 mPa·s/10 s^−1^ in the sham operation group to 4.23 ± 0.42 mPa·s/200 s^−1^, 4.75 ± 0.44 mPa·s/50 s^−1^, and 5.68 ± 2.56 mPa·s/10 s^−1^ in the I/R group, respectively (each *n* = 6, *P* < 0.01; Table 3). DG administration decreased the WBV at shear rates of 200 s^−1^, 50 s^−1^, and 10 s^−1^ to 3.33 ± 0.14 mPa·s/200 s^−1^, 3.75 ± 0.15 mPa·s/50 s^−1^, and 4.50 ± 0.28 mPa·s/10 s^−1^, respectively (each *n* = 6, *P* < 0.01; Table 3). ESR decreased from 0.98 ± 0.15 mm/h in the sham operation group to 0.58 ± 0.06 mm/h in the I/R group. By contrast, in the DG administration group, ESR increased to 1.10 ± 0.24 mm/h (each *n* = 6, *P* < 0.01; Table 3). We also found that Htc did not change (Table 3). These results suggested that DG administration improves hemorheological parameters.

**Figure 4:**
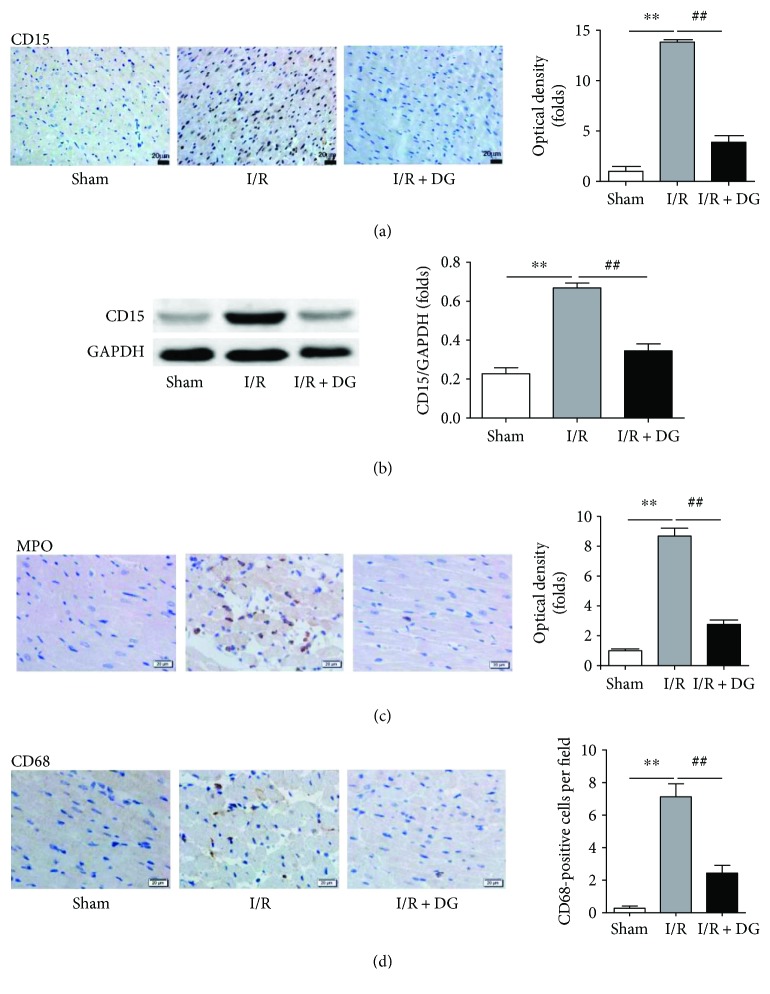
Dunye Guanxinning inhibited myocardial neutrophil infiltration. (a) Immunochemical staining and optical density of anti-CD15 antibody (each *n* = 3); (b) CD15 immunoblot of the left ventricular tissue (each *n* = 4); (c) immunochemical staining and optical density of anti-MPO antibody (*n* = 3); (d) immunochemical staining and the number of anti-CD68-positive cells (*n* = 3). Data are expressed as mean ± SD. ^∗∗^*P* < 0.01 versus sham operation group; ^##^*P* < 0.01 versus ischemia-reperfusion group.

A previous study demonstrated that neutrophil accumulation occurs in response to acute ischemic myocardial injury [21]. Regarding the temporal dynamics of neutrophils, the number of neutrophils was found to immediately increase to a peak value within 3 days [22]. Therefore, to determine the extent of neutrophil infiltration in the myocardium, we used the anti-CD15 antibody to detect the expression of CD15, which mediates the phagocytosis and chemotaxis of neutrophils [23], in section staining and Western blotting. The results of section staining showed that neutrophils accumulated rapidly at 2 h after I/R injury. Moreover, the optical density in the I/R injury group (7882.65 ± 1703.96) was much higher than that in the sham operation group (499.50 ± 272.88, each *n* = 3, *P* < 0.01; Figure 4(a)). DG administration drastically reduced neutrophil accumulation in the myocardium. The optical density in the DG administration group (1918.81 ± 343.96) was significantly lower than that in the I/R group (*n* = 3, *P* < 0.01; Figure 4(a)). The results of Western blotting were similar to those of section staining. CD15 expression in the myocardium was a factor of 3.10 ± 0.80 higher in the I/R group than in the sham operation group. By contrast, CD15 expression was significantly a factor of 1.60 ± 0.52 lower in the I/R + DG group (each *n* = 4, *P* < 0.01; Figure 4(b)). Because myeloperoxidase (MPO) is a key inflammatory enzyme secreted by activated neutrophils and macrophages, and its distribution in ischemic tissues is positively correlated with infarct size [24], we observed its expression in the injured myocardium. Our results indicated that MPO expression in the I/R group was 8.67 ± 0.46-fold higher than that in the sham group (1.00 ± 0.07-fold) and that DG administration reduced the expression of MPO to 2.76 ± 0.29-fold (*n* = 3, *P* < 0.01; Figure 4(c)). However, labeling of macrophages with the CD68 antibody revealed a limited number of macrophage infiltrates in the injured myocardium. Compared with the 0.30 ± 0.13 cells per microscope field (400x) in the sham group, there were only 7.13 ± 0.80 cells per microscope field (400x) in the macrophage infiltrates. However, a difference was observed between the sham and I/R groups, and DG could also reduce the infiltration of macrophages (2.43 ± 0.49 cells per microscope field (400x); *n* = 3, *P* < 0.01; Figure 4(d)). These results suggest that neutrophils were the major inflammatory cells after 2 h of I/R.

**Figure 5:**
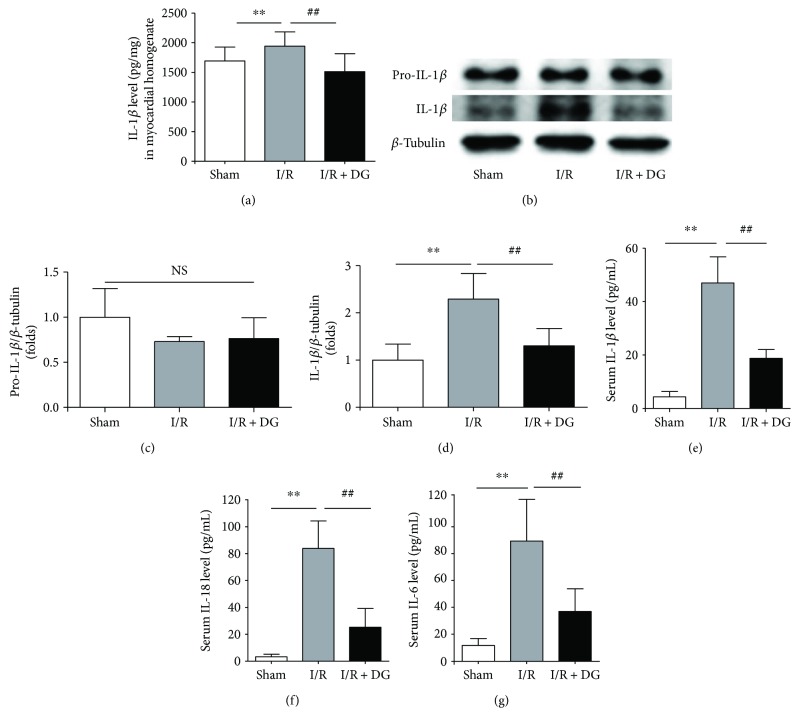
Dunye Guanxinning reduced maturation and release of IL-1*β*. (a) IL-1*β* level in the damaged myocardial homogenate of rats (each *n* = 8); (b–d) Immunoblot levels of pro-IL-1*β* and IL-1*β* in the left ventricular tissue (each *n* = 4); (e–f) serum IL-1*β*, IL-18, and IL-6 levels in rats (*n* = 8). Data are expressed as mean ± SD. ^∗∗^*P* < 0.01 versus sham operation group; ^##^*P* < 0.01 versus ischemia-reperfusion group.

Neutrophil invasion and activation may potentiate the inflammatory response. Therefore, we examined changes in the content of damaged myocardial interleukin-1*β* (IL-1*β*). At 2 h after I/R injury, the IL-1*β* level in the damaged myocardial homogenate of rats was 1948.46 ± 237.04 pg/mg in the I/R group, compared with 1697.97 ± 225.71 pg/mg in the sham operation group. By contrast, this effect was reversed in the I/R + DG group, which had IL-1*β* concentration of 1509.44 ± 302.77 pg/mg (each *n* = 8, *P* < 0.01; Figure 5(a)). This difference in the serum IL-1*β* concentration may be catalyzed by inflammasomes. Thus, we examined the changes in IL-1*β* maturation in the myocardium. Although we found no changes in pro-IL-1*β* expression in the I/R group or the I/R + DG group (Figures 5(b) and 5(c)), IL-1*β* expression in the myocardium was significantly a factor of 2.29 ± 0.54 higher in the I/R group. By contrast, in the I/R + DG group, the expression of IL-1*β* protein in the myocardium was significantly a factor of 1.30 ± 0.38 lower (each *n* = 4, *P* < 0.01; Figures 5(b) and 5(d)). We also measured serum levels of IL-1*β*, IL-6, and IL-18. Serum levels of IL-1*β*, IL-6, and IL-18 in the I/R myocardial injury group (47.06 ± 9.77 pg/mL, 86.59 ± 30.52 pg/mL, and 84.06 ± 19.89 pg/mL, resp.) were significantly higher than those in the sham group (4.36 ± 1.99 pg/mL, 11.32 ± 4.85 pg/mL, and 3.18 ± 1.25 pg/mL, resp.), and DG administration reduced their serum levels (18.59 ± 3.53 pg/mL, 36.08 ± 16.30 pg/mL, and 25.14 ± 13.88 pg/mL, resp.) (each *n* = 8, *P* < 0.01; Figures 5(e)–5(g)). These results demonstrated that DG inhibits neutrophil infiltration and IL-1*β* maturation in the myocardium.

**Figure 6:**
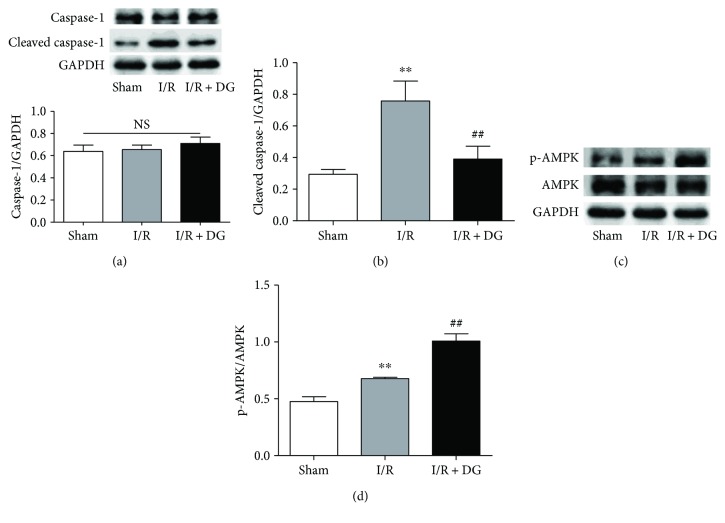
Dunye Guanxinning inhibited cleaved-caspase-1 activity by promoting AMPK phosphorylation. (a–b) Immunoblot levels of caspase-1 and cleaved-caspase-1 in the left ventricular tissue (each *n* = 4); (c–d) immunoblot level of AMPK phosphorylation in the left ventricular tissue (each *n* = 4). Data are expressed as mean ± SD. ^∗∗^*P* < 0.01 versus sham operation group; ^##^*P* < 0.01 versus ischemia-reperfusion group.

Furthermore, to explore the mechanisms through which DG administration reduces IL-1*β* release caused by acute I/R, we examined caspase-1 activity. I/R injury promoted an approximately threefold increase in cleaved caspase-1, and the cleaved caspase-1/GAPDH ratio was 0.29 ± 0.06 in the sham operation group compared with 0.75 ± 0.25 in the I/R group. DG administration inhibited caspase-1 activity and resulted in a ratio of 0.39 ± 0.16 (each *n* = 4, *P* < 0.01; Figures 6(a) and 6(b)). We also found that I/R injury activated the phosphorylation of AMPK, consistent with a previous report [25]. The phospho-AMPK ratio was 0.47 ± 0.09 in the sham operation group but 0.68 ± 0.01 in the I/R group (each *n* = 4, *P* < 0.01; Figures 6(c) and 6(d)). DG administration further improved the phospho-AMPK ratio to 1.00 ± 0.12 (each *n* = 4, *P* < 0.01; Figures 6(c) and 6(d)).

## 4. Discussion

Our results showed that cardiac function was significantly impaired in the I/R group compared with that in the sham operation group. By contrast, the LVEF and LVFS were significantly improved in the I/R + DG group. Furthermore, I/R injury caused a significant reduction in arterial blood flow; however, DG administration resulted in significantly increased carotid arterial blood flow output per minute. In addition, DG administration reduced the myocardial I/R injury infarct area, myocardial apoptosis, and oxidative stress. These findings suggested the high efficacy of DG administration against I/R injury.

Earlier studies have demonstrated that neutrophils participate in the early stages of myocardial I/R injury [21, 26–28]. Our findings showed that DG administration effectively inhibited neutrophil infiltration in the acute myocardial infarction area. Recent studies have demonstrated that myocardial I/R injury causes increased plasma nucleosomes, abundant neutrophil infiltration, and neutrophil extracellular trap formation (NETosis) at the injury site [29]. Moreover, NETosis-mediated microthrombosis was shown to contribute to myocardial “no-reflow” [30]. NETosis may cause chromatin release and extracellular histone accumulation, which induces myocardial cytotoxicity and death and sterile inflammation in the infarcted myocardium [29, 31]. In addition, extracellular histones cause sterile inflammation by activating the NLRP3 inflammasome [32]. Conversely, NLRP3 regulates neutrophil function and contributes to I/R injury [33]. Therefore, DG administration may reduce NETosis, sterile inflammation, myocyte death, and microthrombosis by blocking neutrophil infiltration. Moreover, in the present study, DG administration resulted in increased GSH-Px activity. Early studies have reported that the antioxidant GSH-Px is closely associated with cardiac I/R injury [34, 35]. Early neutrophil infiltration caused by I/R injury induces oxidative stress [36, 37]. This evidence supports that NADPH oxidase and myeloperoxidase are involved in NETosis [38, 39]. Other studies have confirmed that the inhibition of autophagy or NADPH oxidase prevents intracellular chromatin decondensation, which is essential for NETosis [40].

A series of proinflammatory and anti-inflammatory cytokines are well-known to play an important role in myocardial I/R injury. IL-1*β* is one of the most effective early proinflammatory mediators. Previous studies have reported that the ischemic heart exhibited enhanced inflammasome activation, as demonstrated by increased caspase-1 activity and increased IL-1*β* production [41, 42]. It has also been reported that the ischemic heart exhibited enhanced inflammasome NLRP3 expression and caspase-1 activity [43, 44]. Many studies have discovered that IL-1*β* is a critical early inflammatory mediator in myocardial I/R injury, and antagonism of IL-1*β* may have cardioprotective effects [45, 46]. Our results showed that DG administration resulted in significantly decreased myocardial caspase-1 activity and IL-1*β* maturation after myocardial I/R injury. Although studies have revealed that neutrophils and macrophages collaborate to promote IL-1*β* maturation, and cause IL-1*β*-driven and I/R-induced inflammation [47], our results indicated that only a low level of macrophage infiltration occurred in each visual field in the area of myocardial I/R injury at 2 h compared with the sham operation group. Conversely, neutrophil infiltration was substantial. Because neutrophils express key components of inflammasomes and release IL-1*β* and IL-18 proteins [48], it is suggested that DG may be mainly targeted to neutrophil inflammasomes.

Moreover, our results revealed that DG administration activated the phosphorylation of myocardial AMPK during I/R injury. AMPK is a metabolic sensor that coordinates intracellular ATP synthesis and decomposition; thus, AMPK maintains cellular energy homeostasis through the phosphorylation of multiple proteins involved in metabolic pathways [49]. However, the protein abundance and activity of serine/threonine kinase 11, an upstream signaling molecule for AMPK, have been found to be similar in both aerobic and ischemic hearts [25]. Previous studies of our and other groups have demonstrated that ingredients in Chinese medical herbs, such as 2,3,5,4′-tetrahydroxystilbene-2-O-*β*-D-glucoside, bavachalcone, diosgenin, and pterostilbene, stimulated the AMPK activity and anti-inflammatory effects [17, 50–55]. To date, some studies have demonstrated that the activation of AMPK inhibits NLRP3 expression, caspase-1 activity, and IL-1*β* secretion [56–58].

Hemorheological parameters are closely related to hemodynamics, and an increase in low shear blood viscosity is associated with an increased risk of thrombosis [59]. Furthermore, increased red blood cell aggregation is a reflection of inflammation [60, 61]. Moreover, oxidative stress is one of the major factors promoting changes in blood and viscosity [61, 62]. Myocardial I/R injury also causes changes in hemorheological parameters [18–20]. Our results suggested that DG administration improves hemorheology by ameliorating oxidative stress and the inflammatory response.

Our findings revealed that DG administration improved cardiac function, reduced the infarct area, and inhibited cardiomyocyte apoptosis at least partly by inhibiting neutrophil infiltration, activating AMPK phosphorylation, and inhibiting caspase-1 activity and IL-1*β* release during myocardial I/R injury. The findings support the clinical efficacy of DG and partially reveal its mechanism, which is beneficial for understanding the therapeutic effects of the protective traditional Chinese patent drug.

## Figures and Tables

**Table 1:** Saponin content of fresh rhizome of *Dioscorea zingiberensis* C. H. Wright (*n* = 21) [2].

Saponins	Huangjiangsu A	Zingiberensis new saponin	Deltonin	Dioscin	Gracillin
CAS number	1026020-27-8	91653-50-8	55659-75-1	19057-60-4	19083-00-2
Mean ± SD (%)	0.60 ± 0.13	0.50 ± 0.20	0.35 ± 0.11	0.13 ± 0.03	0.18 ± 0.09

**Table 2:** Effect of Dunye Guanxinning on serum CK-MB, LDH, and cardiac GSH-Px activity and GSH content.

Group	Serum CK-MB (U/mL)	Serum LDH (U/L)	Cardiac GSH-Px (U/mg protein)	Cardiac GSH (*μ*mol/g protein)
Sham	0.83 ± 0.72	3365.37 ± 1357.24	320.27 ± 76.48	12.15 ± 5.66
I/R	1.56 ± 0.28^∗∗^	5827.39 ± 1871.24^∗∗^	241.07 ± 76.66^∗∗^	6.84 ± 1.53^∗∗^
DG-treated	0.92 ± 0.29^##^	4112.27 ± 1165.87^##^	327.53 ± 62.91^##^	14.40 ± 10.32^#^

Data are expressed as mean ± SD. *n* = 10. ^∗∗^*P* < 0.01 versus sham operation group; ^#^*P* < 0.05 and ^##^*P* < 0.01 versus I/R group. CK-MB: myocardial creatine kinase isoenzyme; GSH: glutathione; GSH-Px: glutathione peroxidase; I/R: ischemia-reperfusion; LDH: lactate dehydrogenase.

**Table 3:** Effect of Dunye Guanxinning on hemorheological parameters.

Group	WBV (mPa·s)			ESR (mm/h)	Htc (%)
	200 s^−1^	50 s^−1^	10 s^−1^		
DG	3.55 ± 0.18	3.93 ± 0.13	4.70 ± 0.15	1.05 ± 0.04	50.70 ± 0.69
Sham	3.52 ± 0.29	3.88 ± 0.35	4.71 ± 0.48	0.98 ± 0.15	50.86 ± 2.89
I/R	4.23 ± 0.42^∗∗^	4.75 ± 0.44^∗∗^	5.68 ± 2.56^∗∗^	0.58 ± 0.06^∗∗^	48.68 ± 2.34
DG-treated	3.33 ± 0.14^##^	3.75 ± 0.15^##^	4.50 ± 0.28^##^	1.10 ± 0.24^##^	50.48 ± 2.21

Data are expressed as mean ± SD. *n* = 6. ^∗∗^*P* < 0.01 versus sham operation group; ^##^*P* < 0.01 versus I/R group. ESR: erythrocyte sedimentation rate; Htc: hematocrit; I/R: ischemia-reperfusion; WBV: whole blood viscosity.

## Abbreviations

AMPK: Adenosine monophosphate-activated protein kinase
CK-MB: Ceatine kinase isoenzyme MB
DG: Dunye Guanxinning
ECG: Electrocardiogram
ESD: End-systolic diameter
ESR: Erythrocyte sedimentation rate
GAPDH: Glyceraldehyde-3-phosphate dehydrogenase
GSH-Px: Glutathione peroxidase
HRP: Horseradish peroxidase
Htc: Hematocrit
IL-1*β*: Interleukin-1 beta
LDH: Lactate dehydrogenase
LVEF: Left ventricular ejection fraction
LVFS: Left ventricular fractional shortening
NLRP3: NLR family pyrin domain containing 3
SD rat: Sprague–Dawley rat
TTC: 2,3,5-Triphenyltetrazolium chloride
WBV: Whole blood viscosity.
